# Applying Evolutionary Anthropology

**DOI:** 10.1002/evan.21432

**Published:** 2015-02-14

**Authors:** Mhairi A Gibson, David W Lawson

**Keywords:** applied anthropology, behavioral ecology, cultural evolution, development intervention. human behavior, human evolution, public health, social policy

## Abstract

Evolutionary anthropology provides a powerful theoretical framework for understanding how both current environments and legacies of past selection shape human behavioral diversity. This integrative and pluralistic field, combining ethnographic, demographic, and sociological methods, has provided new insights into the ultimate forces and proximate pathways that guide human adaptation and variation. Here, we present the argument that evolutionary anthropological studies of human behavior also hold great, largely untapped, potential to guide the design, implementation, and evaluation of social and public health policy. Focusing on the key anthropological themes of reproduction, production, and distribution we highlight classic and recent research demonstrating the value of an evolutionary perspective to improving human well-being. The challenge now comes in transforming relevance into action and, for that, evolutionary behavioral anthropologists will need to forge deeper connections with other applied social scientists and policy-makers. We are hopeful that these developments are underway and that, with the current tide of enthusiasm for evidence-based approaches to policy, evolutionary anthropology is well positioned to make a strong contribution.

*“An anthropologist's primary duty is to present facts, develop concepts [and] destroy fictions and empty phrases, and so reveal relevant active forces.”*

(Bronisław Malinowski cited in Firth.^1:195^).

Anthropologists have a long history of acting as two-way communicators between local peoples and external global forces. The early goals of anthropology were to provide an explanation of the behavior of unfamiliar and “exotic” peoples, but also to present the native view, highlighting local concerns to administrators and policy-makers.^2^ With the wane of colonialism and the emergence of global communication networks and development aid, the significance of this dual role has continued to grow. Many anthropologists today seek to both identify and communicate the needs of peoples to policy-makers, with the aim of ensuring culturally appropriate and effective forms of development.^3^ Some work as evaluators, examining the successes and failures of specific behavioral interventions, as well as the institutions which run them.^4^ Increasingly, anthropologists focus on important social issues affecting communities across a changing world, such as building resilience to climate change,^5^ urbanization,^6^ sustainable public health,^7^ and food and water security.^8^ In this review, we highlight the work of a growing number of researchers applying an evolutionary anthropological perspective to topics that are relevant to contemporary social and public health policy.^9^,^10^

Recent decades have seen dramatic growth in evolutionary studies of human behavior.^11^,^12^ While not without controversy (Box 1), this growth reflects increasing acknowledgment across the social sciences that evolutionary considerations complement and deepen our understanding of behavioral diversity. We begin by characterizing the theoretical and methodological contributions of evolutionary anthropology and its added value with respect to neighboring social sciences (see also Glossary). Focusing on the themes of production, distribution, and reproduction, we highlight classic and more recent research that is relevant to current efforts to improve human well-being. Throughout, we focus on core strengths of the evolutionary anthropological paradigm, concentrating on insights from naturally observed behavior (rather than stated preferences or experimentally induced behaviors), cross-cultural research, and studies concerning functional (ultimate) explanations. We primarily draw on the tradition of human behavioral ecology, the subfield of evolutionary behavioral science most closely associated with both anthropology and studies of animal behavior.^11^,^13^ We conclude by addressing the challenges and opportunities of applying evolutionary anthropology, along with our own reflections on future research priorities.

**GLOSSARY**

**Ultimate causation** — explanations for behavior grounded in evolutionary history and adaptive function.

**Proximate causation** — explanations for behavior based on underlying mechanisms such as human physiology, psychology, or culture.

**Extrinsic mortality** — causes of death that cannot be mitigated by individual action. The extent to which mortality is relatively extrinsic (largely unavoidable) or intrinsic (largely avoidable) has strong impacts on life-history evolution.

**Life-history theory** — concerns the scheduling and allocation of energy to key factors, such as the timing of reproduction and number of offspring, across the life cycle. Natural selection optimizes trade-offs in resource allocation between competing functions such as reproduction vs. growth, mating vs. parenting effort, and current vs. future reproduction.

**Adaptive lag** — describes situations in which the rate at which an organism adapts is slower than the rate of environmental change, leading to a suboptimal mismatch between behavior and environment.

**Inclusive Fitness** — the sum of direct and indirect fitness. Direct fitness is gained by producing offspring; indirect fitness is gained by aiding related individuals, both lineal and collateral descendants.

**Reproductive success** — a proxy measure for direct fitness, generally measured by the number of offspring surviving to reproductive age (ideally over an individual's life span).

**Optimal foraging theory** — a set of models using optimization methods, based on the principle that selection has designed foraging behavior to maximize the net rate of food acquisition (in some cases conditioned by a measure of risk sensitivity).

**Sexual selection** — a form of natural selection arising from differential mating success. This involves competition with same-sex conspecifics to win mates by force or charm.

**Decision-making mechanism** — means by which environmental information is processed, resulting in the selection of a behavior (or belief) among several alternative possibilities. Decision making is not necessarily conscious.

**Box 1. Common Misunderstandings About Evolutionary Behavioral Anthropology**

**Evolutionary and other social science explanations are not alternatives**. They focus on different levels of explanation. While much of social science deals with proximate-level explanations, most evolutionary anthropologists are interested in whether behavior can be understood in terms of maximizing inclusive fitness or proxies for fitness, such as reproductive success, social status, or energetic return.

**Evolutionary explanations do not make the naturalistic fallacy**. The false belief that what is natural is inherently good or right, and that what is unnatural is bad or wrong. Evolutionary explanations can help us understand the ultimate function of behavior in terms of inclusive fitness, but should not be used to make moral or prescriptive judgments.

**Evolutionary anthropology is not Social Darwinism.** Social Darwinism is the name given to theories which sought to apply the concept of ‘the survival of the fittest’ to social policy in the early 20^th^ Century. Social Darwinism, more properly termed “social Spencerism,” is generally associated with the view that “stronger” members of society should be encouraged to reproduce at the expense of “weaker” members.

**Evolutionary perspectives on behavior do not anticipate that individuals consciously or unconsciously strategize about the fitness pay-offs of alternative behavior.** Instead, natural selection is understood as shaping behavioral motivations toward proximate goals, such as social status, avoiding danger, and obtaining sexual partners, that correlate with fitness.

**Evolutionary anthropologists focus on behavioral diversity.** A common misconception is that evolutionists are interested only in explaining species-typical traits. While some evolutionary anthropology does fall into this category (for example, explanations for menopause), evolutionary behavioral anthropology is primary focused on explaining human behavioral diversity. Indeed, the key to success in our species may be our adaptive behavioral flexibility, enabling us to colonize an unusually wide range of ecologies.

**Evolutionary anthropology is not a form of genetic determinism.** It is a mistaken idea that evolutionists consider genes alone to determine an organism's physiology, behavior, or culture. Rather, it is the interaction of genes and the environment that determines biological and behavioral phenotypes. Evolutionary anthropologists focus on adaptive variation in behavior that allows individuals to adjust their responses to local costs and benefits.

**Figure 1:**
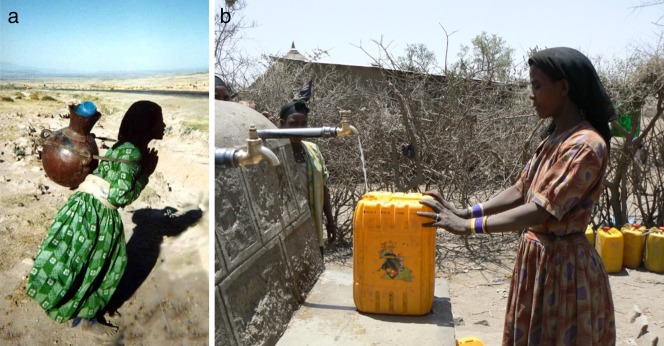
Consistent with evolutionary life-history theory, the arrival of taps, which significantly reduced women's water-carrying loads, has led to higher birth rates in some Arsi Oromo Ethiopian villages (details in Box 2). [Color figure can be viewed in the online issue, which is available at http://wileyonlinelibrary.com.]

## WHAT DOES EVOLUTIONARY (BEHAVIORAL) ANTHROPOLOGY OFFER?

### Theoretical Contributions

Evolutionary anthropology presents a “top-down” research framework in which hypotheses are generated from the theoretical principles of evolutionary biology. This contrasts with the “bottom-up*”* approach more typical of nonevolutionary social sciences, which begin with the description of specific phenomena leading to the incremental generation of theory. Since natural selection, in addition to drift and mutation, is a major driver of evolution, explanations of behavioral diversity typically are framed in terms of evolved responses to problems posed by current and past physical and social environments.^11^–^13^ Thus, while constraints to adaptation are considered, research first and foremost takes an optimality approach, with natural selection understood as shaping the human phenotype in response to the pay-offs from alternative behavioral “strategies.”^14^ Following the sociobiological principles developed by Tinbergen in the early twentieth century and later behavioral ecologists, evolutionary models are unique in considering both ultimate and proximate causation.^15^

Evolutionary anthropology's central premise that behavior can often be understood as functionally tied to the costs and benefits of action, is very close to the principle of utility maximization in economics.^11^ However, it also makes distinctive contributions. Evolution by natural selection not only provides an ultimate explanation of why humans are predicted to behave optimally, but also conceptual clarity with regard to what currency behavior is predicted to be optimizing (that is, its “utility”). Natural selection shapes human behavior and the mechanisms governing it toward maximizing inclusive fitness, or production of long-term genetic descendants. This, however, may come at the expense of physical or mental health, material gain, or other measures of personal or societal well-being.^16^ Understanding this innate susceptibility has been fundamental to studies of “evolutionary medicine,” which views human vulnerability to physical and mental illness as an evolved feature of our biology. This branch of evolutionary studies has built considerable momentum as an applied science and is now being incorporated into medical and public health education.^17^

With an anthropological lens focused on diversity, evolutionary anthropology puts a strong emphasis on ecological contingency. From this perspective, the huge variation in human behavior across and within cultures exists in large part because the pay-offs to alternative behaviors are highly dependent on local circumstance.^11^,^13^,^18^ This standpoint highlights the dangers of ethnocentrism, or the tendency to judge other cultures by the values and standards of one's own. A focus on diversity and contingency also supports arguments for targeted development intervention designed to address local conditions and specific needs, and an *a priori* scepticism of broad-based initiatives applied cross-culturally with little regard for local context. This provides a useful counterpoint to the issue of local-level realities commonly being ignored in development initiatives emanating from and directed by international agencies.^7^

By considering proximate mechanisms of adaptation, evolutionary anthropology can provide us with an improved understanding of the means whereby behavioral strategies are acquired and transmitted, along with expectations regarding departures from optimality. Decision-making mechanisms are understood to be “imperfect” because adaptation works around energetic, developmental, and phylogenetic (that is, historical) constraints, limiting the range of potential behavioral responses. Because natural selection effectively adapts behavior to past, not present, environments, mechanisms of adaptation are predicted to be particularly challenged by environmental novelty resulting from rapid social or ecological change.^19^ However, the speed and extent to which humans are able to adapt to novelty remains a contested issue in evolutionary behavioral anthropology.^12^,^20^

When studying human behavior and its response to socioecological change, it is a useful heuristic to consider three interrelated categories of proximate mechanism, physiological, psychological, and cultural. Responses to the environment are guided by physiological pathways, such as the elevation of adrenaline in response to threat or the suppression of ovulation when a woman is breastfeeding (lactational amenorrhea).^21^ At the psychological level, preferences, motivations, and emotions subconsciously or consciously guide responses to environmental cues such as food availability, psychosocial stress, and mortality risk.^12^,^22^ Studies of cultural evolution consider differential social learning rules, such as tendencies to copy the most frequent behaviors (conformity-bias) or the behaviors of those deemed most successful (prestige bias). Transmitted culture may also introduce new behavioral variants to a population; these may spread and evolve semi-independently of genetic or environmental influences.^23^,^24^ Research of this kind can improve our understanding of the pathways along which behaviors, ideas, and norms are transmitted, and has clear potential to benefit the design of initiatives targeting behavior change. Roberts^22^ and Mesoudi^24^ have recently reviewed the potential applied value of evolutionary studies more focused on proximate accounts of human decision-making.

### Methodological Contributions

As a branch of anthropology, evolutionary anthropology emphasizes the value of culturally appropriate methods, data analysis, and interpretation. It is committed to field work and data collection at the level of specific communities and cultural contexts. This contrasts with evolutionary psychology, in which studies typically are focused on preferences rather than actual behavior, controlled laboratory experiments, and exhaustive use of undergraduate students as the model organism for the study of human behavior (critiqued in Laland and Brown,^12^ Sear, Lawson, and Dickens,^25^ and Henrich, Heine, and Norenzayan^26^). It also differs from other applied sciences, which give priority to data that can be generalized to the majority, such as nation states or administrative zones with limited concern for ethnic, cultural, or subsistence variation. A similar context-sensitive approach to data collection and interpretation is prioritized in biosocial anthropology and anthropological demography.^27^

Evolutionary anthropology also has a strong tradition of mixed-methods research. For any given topic on human behavior, it is customary to encounter research using participatory field work; observational, demographic, and social survey methods; experiments; comparative ethnographic analyses; and formal mathematical modeling. The field has also adapted methods from evolutionary biology, such as the use of phylogenetic comparative analysis, which uses data on historical relationships between populations (incorporating a tree-like history of ethnolinguistic groups) to provide rigorous inferences about cultural change and coevolution, such as explaining changing marital practices and inheritance norms.^28^ This broad toolkit, which continues to expand, including the use of secondary demographic and biomedical datasets^11^ and natural experiments.^29^

## APPLIED THEMES IN EVOLUTIONARY ANTHROPOLOGY

The behaviors studied by evolutionary anthropologists can be roughly categorized into three main interrelated themes: production, distribution, and reproduction.^11^,^13^ We provide a nonexhaustive overview of these thematic clusters, illustrating their relevance to contemporary social and public health policy.

### Production

Human behaviors falling under the category of production include those regarding patterns of resource-use, subsistence, and dietary choices. Production lies at the heart of many global challenges of the twenty-first century, including efforts to achieve sustainable food production^30^ and effective natural resource management.^31^ It is also a central feature shaping the evolution of behavior and life history. Evolutionary anthropological studies of production reveal that social organization, as well as co-operative relationships between the sexes, different generations, kin and nonkin, can all be viewed as co-evolved responses to the demands of producing enough food.^18^ While productive decisions and subsistence behaviors are ecologically contingent, they are also systematic and predictable in nature.^32^

Many evolutionary studies of production employ optimal foraging theory, which focuses on the costs and benefits accrued to individuals from their subsistence or production decisions.^32^–^36^ People are predicted to behave so as to maximize return rates of energy or nutrients and efficiency (per unit of production time); these are viewed as correlates of fitness.^32^ This approach is particularly useful for understanding the effects of changing subsistence practices and diet, including shifts in climate and the availability of new technologies and processed foods.^33^–^37^ For example, among the Cree Ojibwa of northern Canada, the adoption of new technologies has affected hunting in ways that correspond well with optimal foraging theory. The introduction of firearms, which increased the speed and reliability of hunting initially led to an expansion of diet breadth, while the arrival of snowmobiles resulted in improved efficiency in searching for game, reversing this trend toward a narrower diet of one or two species. This study offers clear recommendations for the effective management and protection of marginal forager economies, including introducing game regulations that take into account optimal diet and the encouragement of large home ranges to prevent resource depletion.^33^,^34^

Dynamic models have further extended optimality theory to incorporate the long-term costs and benefits of behaviors, identifying how subsistence decisions may change over time. These studies show that in unpredictable environments, humans may strategically over-exploit natural resources on which they depend in the short term in order to maximize long-term goals.^38^ Much of this work is relevant to wider, often political, debates on conservation and indigenous affairs. In particular, evolutionary anthropologists have actively raised objections to the somewhat romantic view that indigenous peoples are natural conservationists.^39^–^42^

Evolutionary anthropologists and other social scientists are also converging on the idea that subsistence decision-making combines individual strategic goals and needs with those of the wider group.^43^,^44^ In other words, people make subsistence decisions based on shared norms and expectations, as well as individual costs and benefits. This work is relevant to policy on agricultural reform and recent concerns about global food security. Tucker,^43^,^45^ for example, argues that policy-makers have had an extremely narrow focus on profit maximization and a model of farmers as selfish individualistic actors. Developments in cultural evolutionary theory and ethnographic observations suggest that a more realistic model of how people behave should include collective benefits and strategies that avoid extrinsic shocks, such as food insecurity.^45^,^46^ An evolutionary focus has also found that increases in food production may lead to increasing fertility and, consequently, in some instances, exacerbate competition for limited resources (Box 2).

**Box 2. The Impact of Rural Development Technology on Birth Rates in Africa**

Across the developing world, labor-saving technologies are designed and introduced specifically to improve community health and well-being. Evolutionary anthropological studies have been instrumental in demonstrating that such schemes may also have unintended demographic consequences. In a natural experiment provided by the recent arrival of village-level water-tap stands in Arsi Oromo villages in Southern Ethiopia, Gibson and colleagues explored how a reduction in the time and effort women spend collecting and carrying water (Fig. 1) affected the timing of births, deaths, and out-migration in 2,000 households over 15 years. Consistent with predictions from evolutionary life-history theory, Gibson and Mace^77^ demonstrated that the arrival of taps directly led to higher birth rates and shorter birth intervals. In the absence of modern contraception, energy was, in effect, diverted away from work collecting water and into higher birth rates. This indicates a bio-behavioral response to changing energy availability. This response is likely to represent an evolved feature of our reproductive physiology, allowing humans to defer reproduction during periods of energy shortage.^21^ Similar links between new labor-saving technology and increased birth rates have also been identified among Mayan women using grain mills in Mexico.^76^

The Ethiopian study also showed that higher birth rates, combined with increases in child survival due to improved water supply led to larger family sizes and increased resource scarcity within households. The arrival of new taps was associated with higher rates of childhood malnutrition,^77^ increased out-migration, and biases in education within families.^29^ Of relevance to policy-makers, this study demonstrates the need for family planning to be combined with other forms of development intervention. It also supports the argument for community-based, bottom-up rather than vertical, top-down intervention initiatives.^29^

Evolutionary anthropologists and conservation policy makers also share an interest in environmental problems linked to human actions, in particular, the exploitation of common-pool natural resources such as fisheries, forests, grazing lands, fresh water, and fossil fuels.^44^,^47^,^48^ Evolutionary theory provides predictions of the patterns of resource exploitation we are likely to see, but also the extent to which individuals may be more or less inclined to cooperate in order to conserve their environment. For example, people may tend to over-use natural resources because they are motivated to use common pool resources before others do.^47^ However the pay-offs of exploiting these resources may vary not only by age, sex,^49^ wealth, and status,^50^ but also the social and temporal distances of those involved.^51^,^52^ Individual interests may also be constrained by social norms,^43^ population size and composition,^53^ and asymmetries of power and coercion within and beyond the community.^42^ With regard to changing resource-use behaviors, perhaps the most important insights provided by an evolutionary approach is that there is no one set of solutions to all types of conflict over natural resources.^54^ This has practical implications for policy-makers seeking to minimize resource depletion, specifically in being mindful of the full range contextual factors that influence behavior and designing locally appropriate initiatives.

### Distribution

The study of distributive behaviors concerns the allocation of capital to self and conspecifics. Human capital, also referred to as “wealth” or “resources,” can be embodied (incorporated through somatic growth), material (in the form of accumulated goods such as land, cattle, or cash) or relational (via kin or other social networks).^55^ Capital represents both a fundamental determinant and a dimension of well-being. Unsurprisingly then, countless policies and projects aim to encourage behavior that ensures the beneficial distribution of resources and minimizes inequality.

An evolutionary perspective on distribution directs our attention to various factors. Most obviously, altruistic resource transfers are predicted to be particularly high between genetically close relatives.^56^ This prediction is well supported in contexts as diverse as the allocation of care within families,^57^ food sharing in hunter-gatherer bands,^58^ and the international flow of migrant remittances.^59^ Resource transfers are also anticipated to be common in situations of mutual gain and in situations of both direct and indirect reciprocity.^60^ Indeed, the extent of cooperation between nonkin is extreme in humans, leading to a fertile literature about the evolutionary processes at play, revealing the determinants of variation in our cooperative tendencies both within and between cultures.^53^,^61^,^62^

This literature is highly relevant to schemes aiming to redistribute wealth and alleviate poverty, and, more generally, to those seeking to encourage social tolerance and minimize conflict over shared resources. For example, insights on human cooperative tendencies could improve the success of microfinance initiatives, a popular intervention tool in which, for example, self-organizing cooperative groups share responsibility for repaying loans.^62^ Greater understanding of the role of factors such as group size and composition, penalty for noncooperation, and wider features of the social and physical environment could all improve program effectiveness. Such observations are also relevant to the design and maintenance of urban environments. For example, recent evolutionary studies have measured how neighborhood characteristics influence cooperation and antisocial behavior.^63^,^64^

Wealth inequality presents a major contemporary global concern, including rising public awareness and activism with regard to the scale of inequality within Western and economically developing nations. Evolutionary anthropologists are contributing to our understanding of the dynamics of wealth inequality, including its origins, forms, and wider consequences. For example, by compiling comparative data across field sites, a large group of evolutionary anthropologists recently characterized the role of economic systems in determining inequality in somatic, material, and relational capital.^55^ Evolutionary perspectives can also generate hypotheses regarding how inequality may be reinforced by its impact on a range of behaviors. For example, high extrinsic mortality associated with poverty is predicted to further reduce incentives for behaviors that improve well-being in later life, such as exercise, healthy diets, and reduced alcohol or tobacco consumption. Pepper and Nettle^65^ argue that this observation has important implications for public health policy. Most notably, if disinvestment in somatic maintenance represents an adaptive response to risk, then effective interventions are likely to be those that target broader structural changes in the environment. Moreover, “information-giving” policies that seek to educate people about health risks (for example, warnings on cigarette packets) may actually increase disparities in health because the most affluent will be relatively motivated to attend to such information, while the poorest have less incentive to do so.^65^

In other cases, an evolutionary approach sheds new light on the ultimate motivations behind often-counterintuitive patterns of resource distribution among family members within households. This has great potential to inform the design of interventions aiming at safeguarding vulnerable individuals. Hampshire et al.^66^ for example, have reflected on the tension between humanitarian efforts to save the lives of the most disadvantaged children during a severe food crisis in Niger, with local people's need to prioritize long-term household sustainability. Although this study was not explicitly framed in evolutionary terms, it found that parents lack incentives to prioritize the most needful children when overall household survival is more likely to be achieved by allocating food equally or to those offspring most able to engage in subsistence activities. Similarly, Rende Taylor^67^ considered the “dangerous trade-offs” navigated with respect to child sex workers in Thailand, concluding that hazardous labor may be a bearable choice for some parents striving to maintain family property and status. These studies not only suggest that effective interventions require sensitive targeting of specific family members, but also reinforce the rejection of a unitary model of household interests.

Evolutionary studies are addressing the question of how policy may affect distributive behavior. Gibson and Gurmu^68^ explored government-led changes in land tenure in rural Ethiopia to test hypotheses derived from the evolutionary anthropological literature on sibling competition. Harnessing a “natural experiment,” the study confirms the pivotal role of intergenerational wealth transfers in driving male siblings' competition for resources. Only where land is inherited, is a man's marital and reproductive success negatively influenced by the number of his brothers; where land is distributed by the government, sibling relationships are more cooperative. One implication of this study is that land redistribution programs are likely to have far-reaching unintended consequences, not only on family wealth transmission and dilution, but also family relationships and fertility intentions. Shenk^69^ identified the limitations and possible pitfalls of a government ban on dowry in South India, where dowry represents an important form of daughter-biased investment in a male-biased cultural system. Shenk argued that, in this context, an outright ban is unlikely to be effective, since dowry not only provides positive tangible investments in women that help them attract wealthy husbands, but also improve their education and employment opportunities.

### Reproduction

Reproduction, broadly defined here to include behaviors pertaining to sexual and romantic partnership, is closely linked to evolutionary fitness. A substantial evolutionary literature concerns human family systems and relationships between reproductive behavior and well-being.^70^ Reproduction is also a key area of policy concern, most obviously with respect to population growth in the developing world, but also with regard to the design of initiatives to modify patterns of family formation (for example, preventing teenage pregnancy) and mitigate sexual conflicts of interest (for example, by increasing female empowerment). Evolutionary anthropology has the potential to critically inform debates relating to these initiatives and policy targets.

Evolutionary studies of reproduction are often framed in terms of life-history trade-offs between functions such as mating and parenting effort, and investing in offspring quality versus quantity. Economic models of the family share this emphasis on trade-offs,^71^ but an evolutionary perspective uniquely anticipates widespread “tolerated costs” of reproduction. Parents are predicted to sacrifice their own well-being for that of their children. This is in contrast with the view of some demographers that high fertility is motivated by the benefits children bring parents.^71^ Parents are also predicted to sacrifice the well-being of existing children in favor of continued reproduction, provided that doing so maximizes inclusive fitness.^72^ These insights help us understand why people have more children than it seems they can afford and inform us of underlying conflicts of interest between parents and offspring, such as in the feeding practices of young children.^73^

Evolutionary life-history theory also offers predictions about how reproductive behavior will react to environmental change, including change brought about by development intervention. For example, high extrinsic mortality limits the ability to enhance offspring success through increased parental investment^72^,^74^ and, by truncating life expectancy, reduces the returns to delayed reproduction.^75^ Thus, in contexts of unavoidably high pathogen load or inescapable poverty, strategies of high and early fertility are predicted. Human “reproductive ecologists” have demonstrated the pivotal role of energy balance in regulating fertility via pathways such as lactational amenorrhea and the suppression of ovulation under intense energetic stress or nutritional deficit.^21^ Following these observations, improving maternal well-being, for example by reducing workloads. may inadvertently increase fertility and have negative consequences as families struggle to care for additional children^76^,^77^ (Box 2). Programs to improve maternal health may therefore benefit from the integration of culturally appropriate forms of family planning and improved opportunities for parents to invest in existing children through, for example, child health services or education.^29^

Population growth in the developing world is generally viewed as having adverse consequences through increased pressure on public services and infrastructure, a high ratio of young to working-age people, increases in maternal and child mortality, and environmental degradation. Evolutionary anthropologists have contributed to our understanding of the proximate and ultimate drivers of the demographic transition, putting particular emphasis on shifts in the perceived pay-offs to investment in offspring quality over quantity and late over early reproduction as a consequence of declining extrinsic mortality, new payoffs to education, and modern labor markets.^72^,^78^ This literature identifies considerable context-dependency in the costs and benefits of high fertility for parents and children.^72^ Improved understanding of this could provide new insight into the circumstances most likely to stem population growth, as well as how apparent costs of large family size could be alleviated by measures beyond a narrow focus on reducing fertility. Such thinking highlights interesting points of tension among international development targets. For example, while campaigns for universal child education and the abolishment of child labor have clear merit, they may also exacerbate poverty and food insecurity in the short-term by restricting child contributions to productive tasks and assistance in rearing younger siblings.^79^,^80^

Outside of anthropology, most research on family structure is focused on Europe and North America. In these countries, the nuclear family is both the norm and the socially recognized ideal, seriously biasing the current knowledge base available to policy-makers. The large evolutionary literature on alloparenting demonstrates that there is nothing “natural” or intrinsically advantageous about the nuclear family set-up.^81^ By providing data on how the presence of alternative family members influences child outcomes, evolutionary anthropologists have the ability to contribute to a more informed understanding of positive rearing environments.^70^ For example, research emphasizing the importance of extended kin for child rearing is highly relevant to debates regarding the best forms of care for orphaned children, including regions experiencing high adult mortalities via HIV/AIDS.^70^

Sexual selection provides a rich framework for understanding male-female interactions, perhaps most notably where it counters ethnocentric intuitions regarding sex roles.^82^–^84^ Schacht, Rauch, and Borgerhoff Mulder,^84^ for example, call for a reconsideration of a common assumption that more men leads to more violence, with a relative shortage of women encouraging violent competition between men over potential mates.^85^ Drawing on recent theoretical developments in evolutionary biology, they suggest that a male surplus, in certain contexts, may, in fact, lead to lower violence and increased paternal care as males seek to enhance qualities that are attractive to women. Schacht^84^ also argue that further evolutionarily informed investigation may demonstrate the long-term effects of skewed sex ratios at both large scales, such as the highly skewed sex ratios in many Asian countries, and small scales, such as neighborhoods and workplaces. Literature on the evolution of sexual conflict may also provide policy-relevant insights regarding female autonomy and sexual coercion, informing debates on the identification of and appropriate response to violence between intimate partners. Stieglitz et al.^86^ for instance, suggest that among the Tsimane of Boliva, husbands use violence to control women's responses to men, such as engaging in extramarital affairs, which may divert limited resources away from the family. These findings suggest that monitoring men's resource use in marriage, particularly when wages are unstable, may be valuable for public health workers attempting to identify women at risk of domestic violence.

## CHALLENGES AND OPPORTUNITIES

Evolutionary anthropology cannot dictate the goals of social or public health policy. Such concerns are for ethicists; to derive what is “good” from evolutionary theory would commit the naturalist fallacy [Box 1]. Yet by identifying the underlying motivations of behavior, improving our understanding of the nature of human well-being, and identifying conflicts of interest between and within individuals and at multiple scales of analysis, there are many ways in which evolutionary anthropology can inform effective policy. In many cases, it can be used as a means of predicting the consequences of intervention, including those which may be unintended.

We have provided examples in which evolutionary anthropology has clear applied relevance. The challenge now is to turn relevance into action and, for that, evolutionary anthropologists need to forge deeper connections with the traditionally applied social sciences and policy-makers on the ground. Central to this objective is improving communication and collaboration with appropriate decision makers, including national policy makers, research think tanks, and nongovernmental charities. Such people and organizations can help guide our research toward the most pressing human issues and implement our recommendations.

Opportunities to make an impact exist, perhaps more now than ever. There are clear signs that governments, charitable organizations, and social scientists working on the front line of global health and economic development policy are in a reflective mood. Numerous and often controversial books, highlighting the mixed success of international aid and nongovernmental projects have made headlines and bestseller lists in recent years.^87^–^89^ There has also been a spate of articles and books mounting critiques of the tools traditionally prioritized by policy makers in the measurement of physical, mental and socioeconomic well-being, both at the individual and national scale.^90^,^91^ These reviews and critiques all come to a similar set of conclusions. Banerjee and Duflo^88^, in particular, emphasize the need for greater appreciation and understanding of contextual variation in the costs and benefits of alternative behaviors, as well as understanding of the hidden rationality behind the decisions, often seemingly counterintuitive, of disadvantaged people. These points are echoed by Ramalingam,^89^ who notes a need to understand the often fragile equilibria of existing complex systems, stressing the point that well-intentioned but naïve interventions are as likely to exacerbate as to mitigate the problems they address. All of these points are highly consistent with the theoretical and methodological contributions of evolutionary anthropology.

Moreover, there is now widespread recognition that if national and international development policy is to be successful, it needs to be evidence-based, whether through randomized control trials or systematic project evaluation.^88^,^89^,^92^ However, forming such an evidence base is challenging and expensive, particularly for complex interventions. As Ramalingam^89:26-27^ notes *“despite recent pushes for greater scientific accuracy, development and humanitarian work is still not strictly evidence based… there is far more policy-based evidence than evidence-based policy.”* It is our proposition that this evidence gap could and should be met through synergistic exchange with evolutionary anthropology. This exchange can be mutually beneficial. Collaborating with applied social scientists and policy makers provides access to new data and methodologies (such as experimental frameworks), along with relevant expertise and experience. In an age of increased transparency and accountability, such collaborations can also assist anthropologists in meeting demands by funders to demonstrate research impacts on wider society.^9^

To improve uptake of results and establish new partnerships, we also need to disseminate research to a wider audience, through open-access reports, presentations to the public, and non-academic publications. Harnessing the great opportunity of applied evolutionary anthropology will also require shifts in educational practice; the subject is often taught with an exclusive focus on academic rather than applied debates. Some evolutionary anthropologists have undoubtedly been reluctant to address human responses to contemporary issues, given the historical misapplication of evolutionary thought to society (Box 1). An unfortunate product of this resistance is that many outsiders identify anthropology solely with either the relativistic agenda of sociocultural approaches or with the simplistic, ethnocentric forms of evolutionary psychology.^25^ We encourage evolutionary anthropologists actively to redress this balance by considering how research insights may improve human welfare and encouraging students and junior researchers to stay well informed on contemporary world issues. We hope that this review itself succeeds in addressing this final recommendation, stimulating further research and teaching, and encouraging dialogue on applied topics.

## PRIORITIES FOR FUTURE RESEARCH

Opportunities for greater exchange with policy-makers can also be achieved by directing research toward priority areas. To this end, we identify several developments that we believe would be most valuable.

Evolutionary anthropology traditionally has given priority to the study of populations most similar to our evolutionary past (foragers and small-scale, high-fertility, high-mortality subsistence economies). A focus on nonindustrial populations has proved useful for testing evolutionary predictions about human behaviors; it has provided important insights into how our ancestors lived and the processes underpinning important behavioral shifts across human history, such as the agricultural revolution. However, evolutionary anthropologists increasingly are turning their attention toward communities that are on the cusp of transition, including market-integrated and industrialized economies,^11^ and those experiencing the effects of recent population growth, urbanization, and climate change. Focusing on contemporary communities in transition enables us to develop a clearer understanding of important and often controversial issues in evolutionary behavioral anthropology, among them adaptive lag, decision-making in uncertain environments, and the dynamics of behavioral and cultural change. Furthermore, transitional populations typically are those that face the greatest social and health challenges, linked with rising inequality and growing demands for food, employment, and public services.

One area where the international development community is clearly lacking in anthropological expertise is current efforts to intervene in so-called “harmful traditional practices,” a term used to describe practices of non-Western cultures that are deemed detrimental to the well-being of individuals, particularly women and dependents. Evolutionary anthropologists have studied a range of cultural practices that are often given this label, including polygynous marriage,^70^,^93^ infanticide,^94^ genital mutilation,^95^ child marriage and early motherhood.^96^,^97^ and domestic violence.^86^ We encourage evolutionary anthropologists to further existing attempts to understand these behaviors and to communicate their findings widely. An evolutionary approach may be useful in revealing the divergent or overlapping interests of those involved in such practices; clarify the true extent to which they should be considered harmful, and to whom; and help design culturally appropriate interventions. In some cases, they also offer academically intriguing topics and “evolutionary puzzles” as potential examples of maladaptive behavior.

While this review has focused on functional approaches in evolutionary behavioral anthropology (that is, human behavioral ecology), we support further development of applied mechanistic approaches, particularly the study of cultural transmission. A more grounded theorization of culture is badly needed in the applied social sciences, where culture is routinely presented as an explanation, but rarely defined, measured, or directly evaluated as a plausible determinant.^24^,^98^ Furthermore, studies of social learning mechanisms may greatly improve our understanding of the dynamics of behavior change at both individual and group levels. To date, much of the literature on cultural evolutionary processes can be criticized for overreliance on formal mathematical modeling and a relative lack of empirical research.^25^ Therefore, perhaps the most promising are studies that aim to explore the dynamics of social learning in the field and empirically test alternative hypotheses on, for example, cooperation^99^ or contraceptive uptake.^100^

## EVOLUTIONARY ANTHROPOLOGISTS NEED ONLY APPLY

Evolutionary anthropology provides a useful integrative “top-down” theoretical framework linking ideas about the causes of behavioral variation to current debates concerning its consequences for human well-being. It also contributes new sources of data and mixed methodologies, particularly by its dedicated focus on field work within well-described cultural contexts. We hope we have successfully demonstrated these contributions of evolutionary anthropology and their relevance to contemporary public health and social policy. We conclude by emphasizing a final strength of evolutionary anthropology: its acute focus on empiricism and robust capacity to adapt to new theoretical and methodological developments. The primary value of the evolutionary anthropology paradigm is not its marriage to a particular set of assumptions, but rather its commitment to asking complicated (ultimate and proximate) questions about why people do what they do and empirically evaluating alternative hypotheses. Not doing so is to rely on rhetoric and implicit assumptions about human nature. We propose that evolutionary anthropology holds great promise, not only to increase our understanding of the world, but also to improve it. With the current wave of enthusiasm for evidence-based approaches to policy and shifts in research dissemination and practice, we believe it is well positioned to make a strong contribution. Evolutionary anthropologists need only apply.
